# Effect of administration of nanoparticles of human chorionic gonadotropin on testicular hemodynamics, testicular volume, testicular echotexture, and circulating testosterone and nitric oxide in pubescent goat bucks under heat stress conditions

**DOI:** 10.1007/s11259-024-10634-3

**Published:** 2025-01-15

**Authors:** Mayada Essam, Mohamed A. I. ElSayed, Fady Sayed Youssef, Haney Samir

**Affiliations:** 1 Department of Theriogenology, Faculty of Veterinary Medicine, Cairo University, Giza, 12211 Egypt; 2 Pharmacology Department, Faculty of Veterinary Medicine, Cairo University, Giza, Egypt

**Keywords:** HCG, Nanoparticles, Testicular hemodynamics, Echotexture, Nitric oxide, Testosterone, Estradiol, Total antioxidant capacity, Goats

## Abstract

The current study investigated the effect of a single administration of human chorionic gonadotropin hormone (hCG) and its nanoparticles (NPs) on testicular hemodynamics using Doppler ultrasonography, testicular volume, testicular echotexture (PIX), and circulating testosterone and nitric oxide (NO) in pubescent goat bucks during summer months. Fifteen Baladi goats were divided into three groups (5 in each) and subjected to a single intramuscular administration of one ml of physiological saline ( control group), one ml containing 500 IU of hCG (hCG group) or one ml containing 125 IU of hCG NPs (hCG NPs group). Testicular hemodynamics assessment was done just before administration (0 h), and at 2, 4, 6, 24, and daily till 7 days after administration. Testicular volume and PIX were measured and analyzed by B-mode ultrasonography and computer analysis software. Testosterone and NO concentrations were measured using commercial kits. Results revealed significant decreases (*P* ˂0.05) in Doppler indices values (resistance index and pulsatility index) especially at 24 h in the hCG group (0.314 ± 0.01, 0.382 ± 0.01, respectively) compared with the hCG NPs group (0.428 ± 0.01, 0.567 ± 0.02, respectively), and the control group (0.464 ± 0.006, 0.64 ± 0.01, respectively). There were significant increases (*P* < 0.001) in testicular volume, PIX, testosterone, and NO concentrations in hCG NPs and hCG groups compared to the control group. There were no significant differences between the hCG NPs and hCG groups (P ˃ 0.05) in testicular volume and PIX, while testosterone significantly increased in the hCG group compared to the hCG NPs group and NO was significantly increased in the hCG NPs group compared to the hCG group. In conclusion, a single administration of hCG or its NPs improved testicular vascularization, testicular volume, and circulating testosterone and NO in pubescent goat bucks. So, hCG NPs could be recommended for improving the reproductive performance of goat bucks under heat-stress conditions.

**Supplementary Information:**

The online version contains supplementary material available at 10.1007/s11259-024-10634-3.

## Introduction

Goats are one of the important livestock in Egypt because of their contributions as a potential source of meat and milk production, and goat cheese as a by-product (Marai et al. 2002). Baladi goat is the most common breed reared in the Nile Delta, Egypt. They are a non-seasonal breed and reach puberty at 4 to 6 months (Hashem and Hammam 2009). There is a need to enhance the productivity of goats based on improving the fertility potentials of both males and females. Indeed, the selection of males with high reproductive performance capacity is crucial in small ruminant herds (Camela et al. 2019). Assessment of male fertility potential is addressed by breeding soundness evaluations (BSE). BSE includes physical examination (Maquivar et al. 2021), external and internal genitalia examination (Tibary et al. 2018), scrotal circumference estimation (Maroto-Morales et al. 2016), and assessment of different parameters of semen quality evaluation (Tibary et al. 2018). Doppler ultrasonography is one of the reliable techniques for estimating male fertility potential by assessment of the testicular hemodynamics (Gloria et al. 2020; Ribeiro et al. 2020). Doppler ultrasonography is a helpful noninvasive technique for evaluating the blood flow within the testicular arteries in various animal species (Hedia et al. 2019; El-Sherbiny et al. 2022a). Doppler indices (resistance and pulsatility indices; RI and PI, respectively) are widely employed in clinical studies of testicular blood flow (TBF) in animals under physiological and pathological circumstances (Günzel-Apel et al. 2001; Gumbsch et al. 2002; Pozor and McDonnell 2004; Samir et al. 2020). Several studies have reported strong relationships between Doppler indices (RI and PI) of TBF and testicular functions (Gloria et al. 2020; Samir et al. 2020). Decreased RI and PI values indicate an increase in the testicular vascular perfusion, which is useful for a continuous supply of nutrients and oxygen to the testis (Dickey 1997; Ginther 2007; Varughese et al. 2013; Bollwein et al. 2016). The increased TBF corresponds to the increase of testosterone levels and testicular volumes, improves some parameters of semen quality, and also boosts overall fertility (Strina et al. 2016; Samir et al. 2020).

In males, normal testicular function and spermatogenesis are sensitive to temperature as thermal stress has negative effects on all major cells within the testis resulting in lower sperm quality and quantity characteristics. Due to this reason, in most mammals, the testes are located outside the body in the scrotum to regulate the intratesticular temperature around 32 °C (slightly lower than the body’s core temperature) for normal spermatogenesis (Setchell 2006; Paul et al. 2008; Hansen 2009; Shahat et al. 2020). Heat stress during the non-breeding season (summer months) can induce oxidative stress conditions, which may contribute to the reduction in reproductive capacity (Lu 1989). The overproduction of free radicals (superoxide anion radicals, hydroxyl radical, hydrogen peroxide, and singlet oxygen), which are continuously produced during normal aerobic metabolism (Bernabucci et al. 2002) decreases the TBF (Hedia et al. 2020). TBF is the main pathway for nutrients, oxygen, and other regulatory hormones to and from the testes and a stable blood supply is required for its function and maturation (Setchell 1990). So, any reduction in TBF may cause ischemic damage to the testis, which impairs spermatogenesis, lowers the reproductive performance (Kay et al. 1992), and affects testicular hemodynamics, semen quality, and fertilizing capacity (El-Sherbiny et al. 2022b). Due to the adverse effects of oxidative stress on testicular blood perfusion, testosterone production, sperm quality, and fertility (Bansal and Bilaspuri 2011; Hedia et al. 2020), several studies were conducted to mitigate the negative effects of heat stress on testicular functions using hormones (gonadotrophin) in goat bucks (Samir et al. 2015; Abbas et al. 2021), antioxidants (zinc sulfate-folic acid combination and L-carnitine) in rams (EL-Sherbiny et al. 2022c; Fadl et al. 2022), and amino acids (betaine) in goats (Dangi et al. 2016).

Human Chorionic Gonadotrophin (hCG) is a glycoprotein hormone secreted by a pregnant woman’s placenta, which stimulates the corpus luteum’s secretion of progesterone during the early stages of pregnancy, and plays a role in placentation via sustaining the uterine vasculature’s angiogenesis, and encourages the cytotrophoblast differentiation into syncytiotrophoblasts (Shi et al. 1993; Rao and Alsip 2001; Zygmunt et al. 2002). In veterinary practices, it is used as a valuable diagnostic assay for testing the functional capacity of the male reproductive endocrine system (Samir et al. 2015) as stimulating the Leydig cells to produce testosterone upon its administration (Altoé et al. 2014) as reported in several animal species such as in equine (Pozor et al. 2006) and bulls (Abdelnaby 2022).

Administration of hCG may participate in the regulation of testicular blood perfusion by its high affinity to stimulate angiogenesis via stimulating vascular endothelial growth factor (VEGF) secretion (Papparella et al. 2013). Human Chorionic Gonadotrophin may increase the testicular blood perfusion by a direct effect on testicular vessels via induction of symptoms like inflammation and inhibition of vasomotion with precapillary sphincters relaxation within 1 h (hr) after injection in rats (Bergh et al. 1987). It increased testosterone levels and TBF in stallions (Pozor et al. 2006; Bollwein et al. 2008) and increased the testicular volume in men (Kim et al. 2011), rats (Papparella et al. 2013), and goat bucks (Samir et al. 2015).

Recently, systems of drug delivery based on nanotechnology have been used for improving drugs, hormones, and their biological activity. Nano-hormone delivery systems provide many benefits such as decreasing the dosage of the hormone, which in turn lowers the cost and provides an enhancement of the pharmacokinetics and pharmacodynamics of many hormonal treatments. For example, nano-GnRH improved artificial insemination outcomes of rabbits (Hassanein et al. 2021) and the luteal function of pregnant goats (Hashem and Sallam 2020) even with a reduction of the dose. In study, the dose was reduced to three- to four-fold the conventional dose without affecting the fertility in goats (Hashem and Sallam 2020). Another example is chitosan nanoparticles of hCG, which are provided as a nasal spray, increasing the induction of ovulation in dairy cattle (Pamungkas et al. 2016). Overall, we hypothesized that hCG administration, in its nanoparticle forms, could be beneficial to combat the adverse effects of heat stress conditions on testicular function in male goats by increasing TBF, testosterone levels, and testicular volume. Therefore, the objective of this study was to investigate and compare the effect of hCG and hCG nanoparticles (hCG NPs) on testicular hemodynamics, testicular volume, testicular echotexture (PIX), and circulating testosterone and nitric oxide (NO) in pubescent goat bucks under heat stress conditions (during summer months).

## Materials and methods

This study was conducted according to the guidelines and regulations of the Animal Ethical Committee belonging to the Faculty of Veterinary Medicine, Cairo University, Egypt (Vet CU 25122023889).

### Animals and management

This study was performed at Theriogenology Department, Faculty of Veterinary Medicine, Cairo University (30.0154° N, 31.2120°). Fifteen Baladi goat bucks (*Capra hircus*) weighing 10–15 kg, and 4–6 months- old, were used in this study during August 2023. Goat bucks were kept indoors and received their nutritional requirements based on NRC recommendations with clean water availability *ad* libitum. Each goat buck was exposed to routine clinical examinations including complete ultrasonographic examinations of the reproductive tract to exclude the presence of any abnormalities before starting the research. Goat bucks were routinely vaccinated and dewormed against endemic diseases in Egypt.

### Assessment of heat stress

According to the Egyptian meteorological authority, temperature measurements (T) and relative humidity (RH) records were obtained for the trial period. The average T, RH, and Temperature Humidity Index (THI) during the study were 33 ± 0.26 °C, 61.06 ± 1.26%, and 84.25 ± 0.50, respectively. The daily thermal amplitude (DTA ) was 7.75 ± 0.25, ( which was narrow DTA). To determine whether the studied bucks were experiencing heat stress or not, THI was used according to a formula previously published (Kendall and Webster 2009; El-Tarabany et al. 2017): THI = (1.8 × T + 32) − [(0.55 − 0.0055 × RH) × (1.8 × T − 26)]. This allowed for the determination of the buck’s three stress levels: not stressed (THI < 70), moderately stressed (THI = 70–80), and severely stressed (THI > 80). Therefore, based on this data, the study’s bucks experienced severe heat stress since the THI was 84.25 ± 0.50.

### Preparation of NPs of hCG hormone

Detailed procedures for the preparation and characterization of hCG NPs are illustrated in Supplement File 1. In brief, materials used in the formulation of NPs of hCG were lyophilized hCG hormone powder (water phase), corn oil (oil phase), surfactant (anionic sodium dodecyl sulfate, SDS), and Ultrasonic probe system (1000 W high power) (Scientz-II D; Ningbo Scientz, Zhejiang, China).

#### Aqueous phase preparation

In phosphate-buffered saline (pH 7.4), hCG hormone was dissolved to obtain a solution under sterile conditions. SDS surfactant was added dropwise into the aqueous solution under magnetic stirring at 500 rpm for 30 min to obtain a stable hCG-surfactant system.

#### Nanoemulsion preparation

The oil phase was prepared by adding the required volume of corn oil to achieve a 1:10 volume ratio between oil and water under optimized conditions. The aqueous hCG and oil phases were mixed using magnetic stirring for 15 min to obtain a coarse emulsion.

#### Ultrasonication

The coarse emulsion was further sonicated using an ultrasonic probe at 60% amplitude and one second on-off pulse. Total sonication time was varied from 5 to 15 min to determine optimum droplet sizes.

#### Isolation and drying

The prepared hCG nanoemulsion was centrifuged at 20,000 rpm speed to sediment the hCG NPs formed. The supernatant was discarded, and NPs were vacuum-dried overnight at −20 °C to retain hormone bioactivity.

### Experimental design

**Fig. 1:**
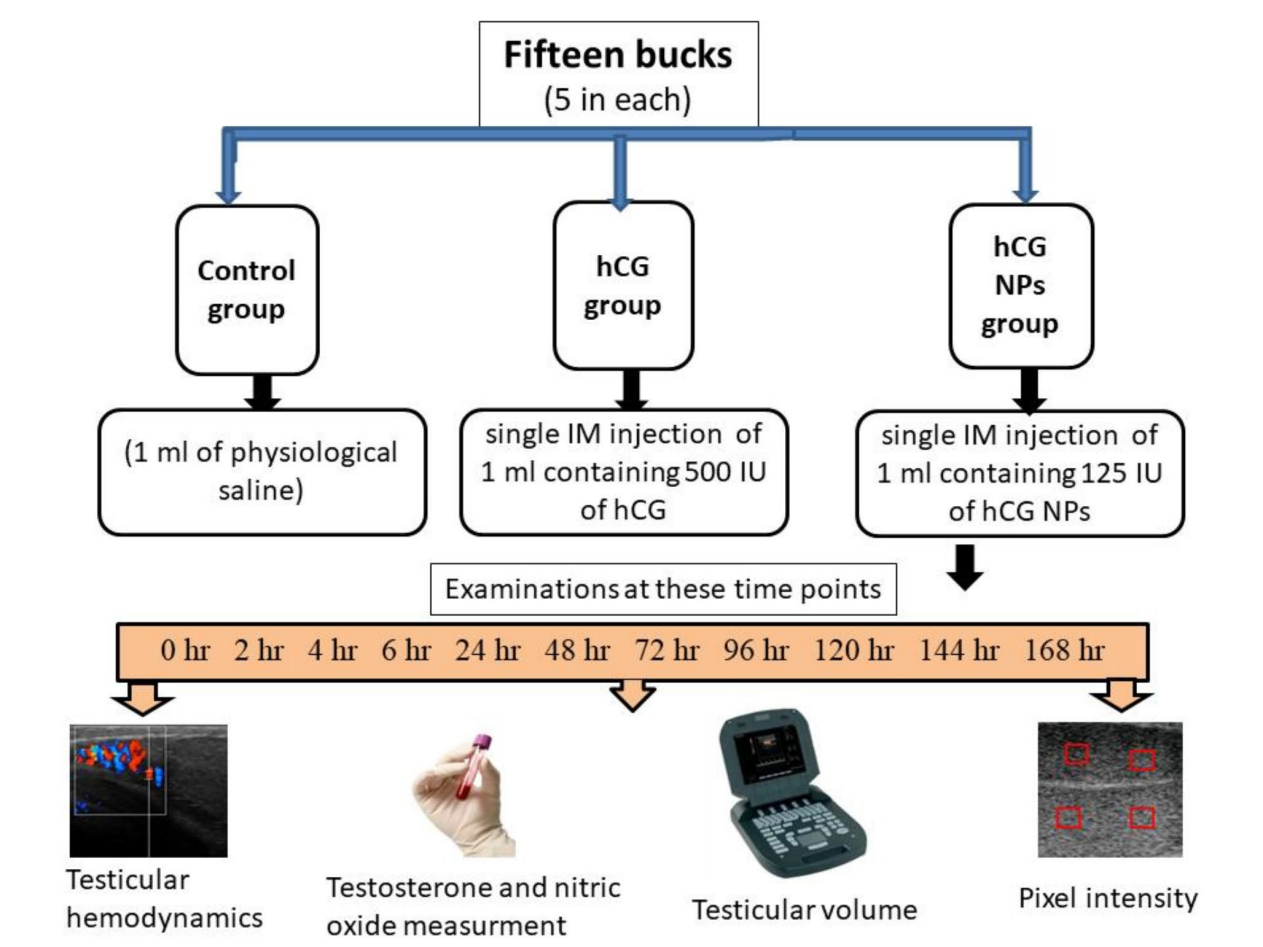
Schematic diagram of the experimental design

All experimental procedures are illustrated in Fig. 1. Goat bucks were divided into 3 groups (5 goat bucks, each): (1) the control group, they were administered a single intramuscular injection of 1 ml of physiological saline, (2) the hCG group, which was administered a single intramuscular injection of 1 ml containing 500 IU of hCG (Epifasi 5000 IU; Eipico Co., Giza, Egypt). This ampoule was diluted by 10 ml of saline before injection. The dose was chosen based on previous studies in goats (Saleh et al. 2012; Samir et al. 2015), (3) the hCG NPs group, they were administered a single intramuscular (IM) injection of 1 ml containing 125 IU of hCG NPs in the form of emulsion (Hashem and Sallam 2020). Each buck has been examined for testicular artery hemodynamics parameters (RI and PI) in the supra testicular artery (STA), PIX, and testicular volume at different time points [at 0 h (just before injection as baseline), and 2, 4, 6, 24, and daily till 7 days post administration in both groups]. Blood samples were collected from the jugular vein at the same time points of ultrasonographic examination to assess the changes in the circulating testosterone and NO metabolites in the serum.

### Blood sampling

Blood samples were collected from the jugular vein of each buck at each ultrasound examination in plain tubes (Vacutest; Shandong Yaohua, China). Three ml of blood was withdrawn at 0 h (just before injection as baseline), and 2, 4, 6, 24, 48, 72, 96, 120, 144, and 168 h after injection in both groups. All blood samples were centrifuged at 3000 rpm for 15 min then the serum was separated and stored at − 20 °C until further analysis. These times were selected based on the pharmacokinetics of hCG and previous studies in goats (Saleh et al. 2012; Samir et al. 2015).

### Hormonal and biochemical analysis

Testosterone concentrations (ng/ml) were measured in serum samples by using a commercial ELISA kit (Diasino Laboratories Co., Ltd, China) with an assay sensitivity of 0.055 ng/ml and inter-precision *≤* 20%. One of the oxidative biomarkers that was assayed in this study was NO metabolite concentrations. Concentrations of NO metabolites were measured calorimetrically by using a commercial kit (Nitric oxide kit, Bio-diagnostic Co., Dokki, Giza, Egypt) using a spectrophotometer at 540 nm wavelength. The assay sensitivity was 0.225 µmol/l in nitrite form and intra- and inter-assay coefficient variations were 5.3 and 6.9%, respectively.

### Ultrasonographic examinations

All ultrasonographic examinations were done by the same operator using the ultrasound scanner (EXAGO, Echo Control Medical, IMV, France) provided with a 5–7.5 MHz linear probe. Before starting the ultrasonographic examinations, each animal was restrained in the lateral recumbency position with the aid of assistance without any sedation, and the hair on the scrotum till the spermatic cord was removed carefully without injuries via shaving. An adequate amount of ultrasonic gel was added to the transducer eliminating air spaces to facilitate the ultrasonographic assessment.

#### Testicular volume and echotexture assessment using B-mode ultrasonography

For testicular volume estimation, each testis was assessed gently by B-mode ultrasonography without pressure until the mediastinum testis appeared. Longitudinal and cross-section images of each testis were recorded. The testicular length (L), width (W), and height (H) were measured using electronic calipers from the frozen B-mode images. These measurements were used for calculating the testicular volume according to the following formula (Samir et al. 2015):

$$\text{testicular volume (ml)} = \text{L}\times \text{W}\times \text{H}\times 0.71$$

For assessment of the echotexture of the testicular parenchyma, each testis was imaged in longitudinal and cross sections for further analyses using computer-assisted image analysis software (Adobe Photoshop CC program) as previously described (Ahmadi et al. 2012; Brito et al. 2012; Pozor et al. 2017). For the longitudinal sections, the transducer was put on the lateral side and parallel to the longitudinal axis of the testis, and for the cross-sections, the transducer was perpendicular to the longitudinal axis of the testis (Samir et al. 2018). Four rectangular-shaped areas were chosen and selected on saved images of homogeneous testicular parenchyma above and below the mediastinum for each testis separately excluding any artifact in the images. The left and right testes results were averaged for analysis.

#### Testicular hemodynamics evaluation

**Fig. 2:**
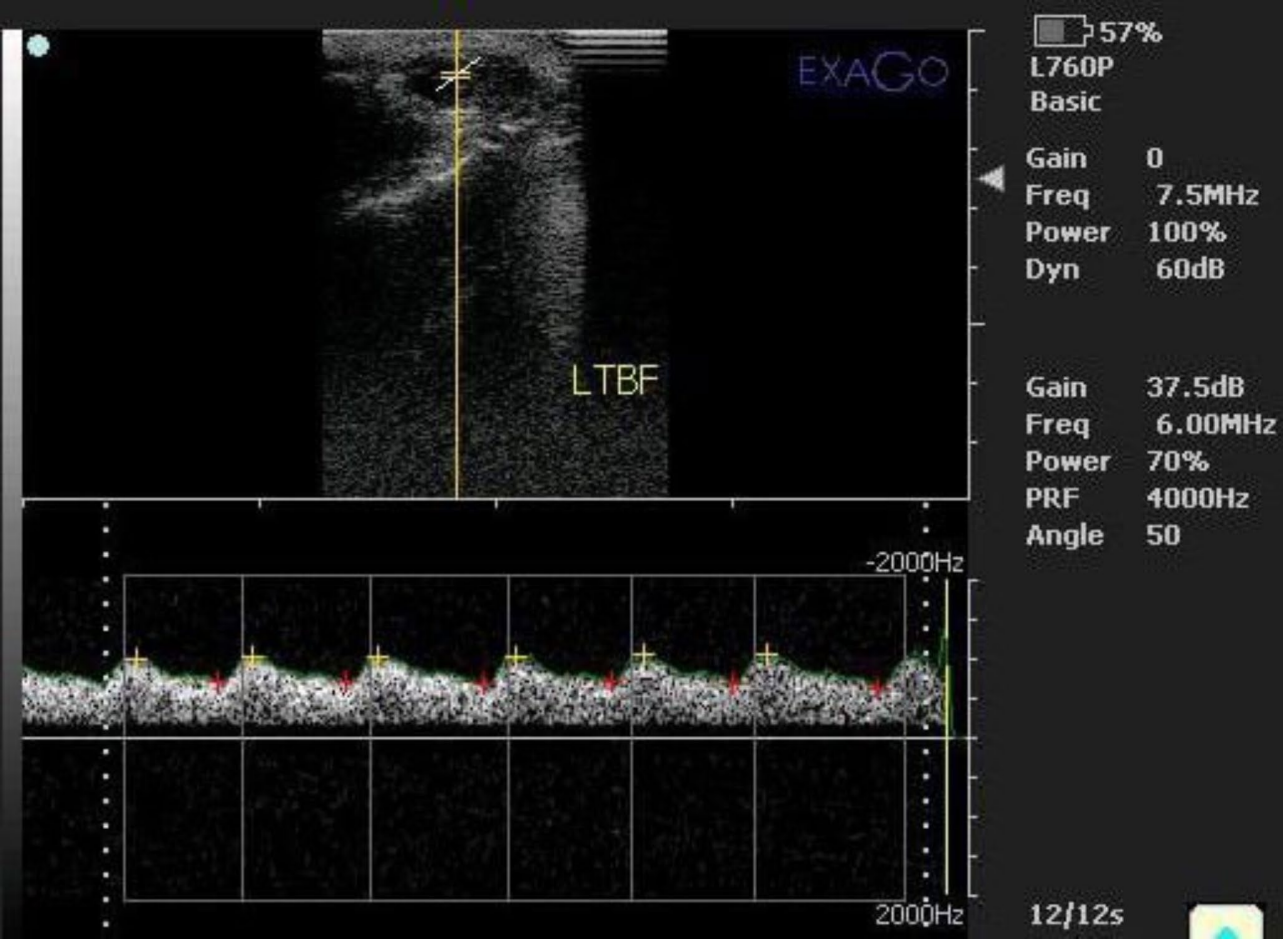
The spectral pattern of the STA using color-pulsed Doppler ultrasonography

To assess the hemodynamic changes within the STA, the transducer was placed vertically on the proximal pole of the testis and then moved upward till the appearance of the vascular network including the STA intertwined with the testicular veins in the pampiniform plexus by using B-mode ultrasonography. Then after, the color and pulsed Doppler modes were activated to visualize the waveforms of blood flow within the examined segment of the STA (Fig. 2). Three to -five successive waves with complete systolic and diastolic cardiac cycles were measured for assessment of Doppler indices as the RI, PI, and systolic/diastolic ratio (S/D ratio) (Gumbsch et al. 2002). All spectral-Doppler settings including focus, brightness, contrast, and gain were maintained fixed for all examinations. The angle between the long axis of the examined vessel and the Doppler beam was less than 60 degrees in the direction of blood flow (Fig. 2).

### Statistical analysis

All statistical analyses were performed by GraphPad Prism 5 software. The normality of the data was examined by the Kolmogorov-Smirnov test. All data in this study were normally distributed and expressed as the mean ± standard error of the mean (SEM). The differences between control, hCG, and hCG NPs groups, in terms of testicular volume, PIX, TBF (RI, PI, and S/D ratio), testosterone, and NO concentrations, and throughout the studied time points (from 0 to 168 h; time effect) and combined treatment time effect were analyzed using two-way ANOVA test. A probability value of at least < 0.05 was considered significant. The differences between the right and left testis were non-significant; therefore, pooling of data of both testes was done and expressed per buck.

## Results

### Testicular hemodynamics

**Fig. 3:**
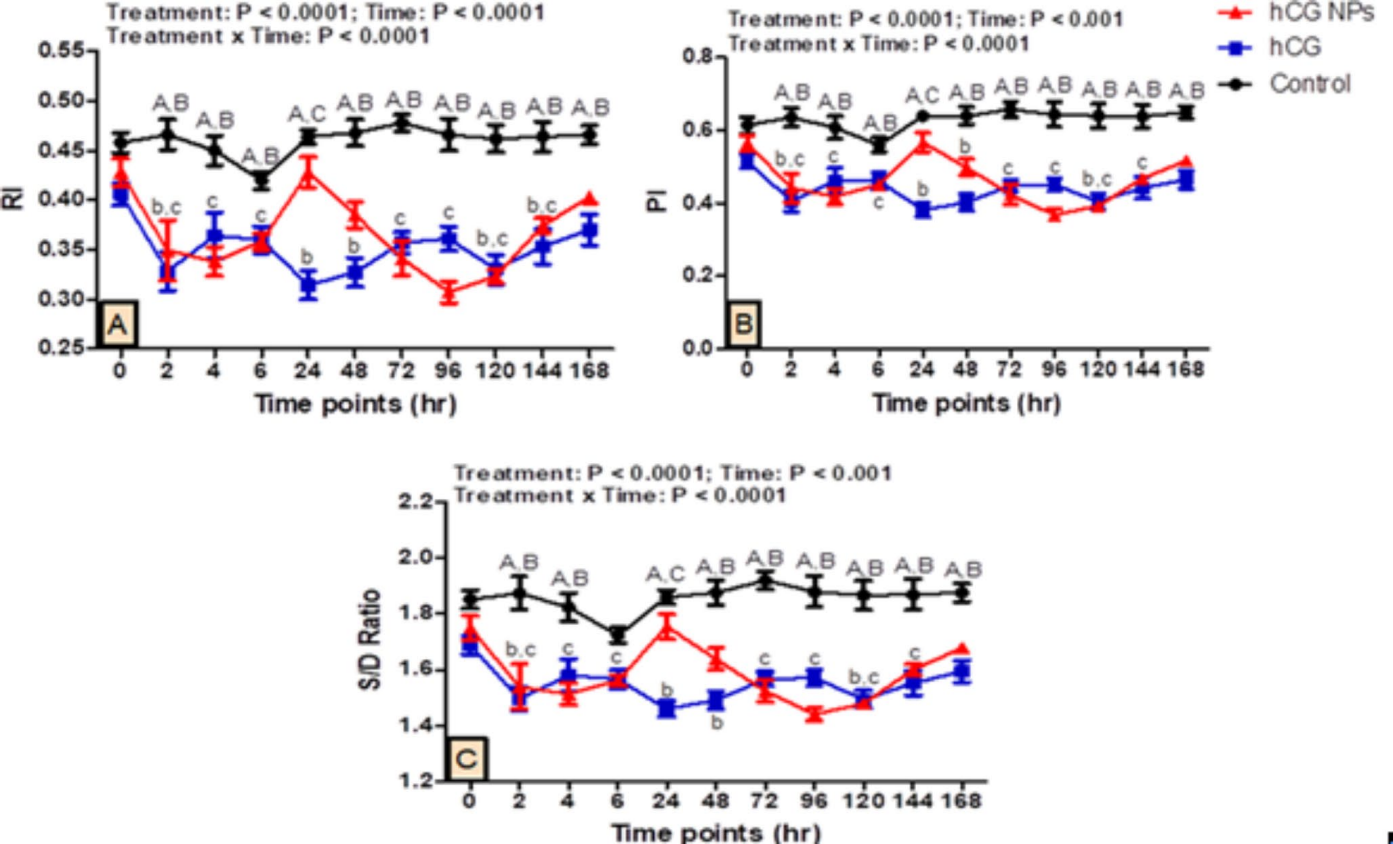
Changes in the parameters of testicular hemodynamics [RI (**A**), PI (**B**), and S/D ratio (**C**)] at the level of supratesticular artery as measured by pulsed-wave Doppler ultrasonography in pubescent goat bucks in the hCG NPs group (red line), hCG group (blue line), and the control group (black line) (*n* = 5, each) during different time points (hours). Values are means ± SEM. Values with superscripts A, B, and C represent significant differences between groups of hCG and control, hCG NPs and control, and hCG NPs and hCG, respectively, during the time point of the study (*P* < 0.05 for all). Values with superscripts b, and c represent significant differences between 0 h and the indicated point within the hCG and hCG NPs group, respectively

The effect of hCG NPs and hCG administration on testicular hemodynamics in pubescent goat bucks is presented in Fig. 3. There were treatment, time, and their interaction effects (*P* < 0.0001, *P* < 0.001, and *P* < 0.0001, respectively) on the values of RI, PI, and S/D ratio. Compared to the control group, the hCG NPs and hCG groups had lower RI, PI, and S/D ratio values at almost all time points of the study.

The RI, PI, and S/D ratio values, especially at 24 h, were significantly lower in the hCG group (0.314 ± 0.01, 0.382 ± 0.01, 1.46 ± 0.02, respectively) compared with the hCG NPs group (0.428 ± 0.01, 0.567 ± 0.02, 1.755 ± 0.04, respectively, *P* < 0.001).

### Testicular volume and echotexture

**Fig. 4:**
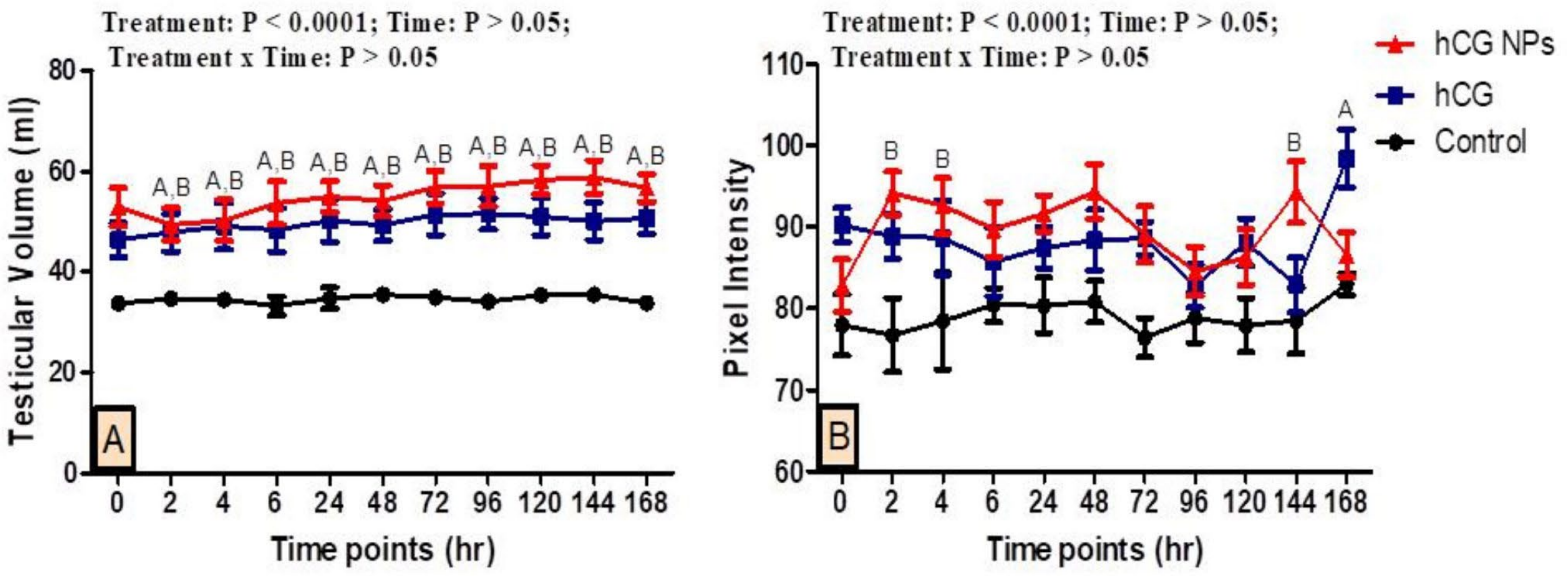
Changes in the testicular volume (**A**) as measured by B-mode ultrasonography and testicular echotexture (**B**) [pixel intensity; PIX] as measured by computer analysis software in pubescent goat bucks in the hCG NPs group (red line), hCG group (blue line), and the control group (black line) (*n* = 5, each) during different time points (hours). Values are means ± SEM. Values with superscripts A and B represent significant differences between groups of hCG and control and hCG NPs and control, respectively, during the time point of the study (*P* < 0.05 for all)

The effect of hCG NPs and hCG administration on testicular volume and PIX is presented in Fig. 4. There were treatment effects on the values of testicular volume and PIX (*P* < 0.0001). While time and interaction (treatment x time) effects showed non-significant effects in values of testicular volume and PIX (P˃0.05). In addition, there was a significant increase (*P* < 0.05) in testicular volume values at all studied time points in the hCG NPs and hCG groups compared to the control group, while there were no significant differences between both hCG NPs and hCG groups (P ˃ 0.05). There was a significant increase (*P* < 0.05) in PIX value at 2, 4, 144 h in the hCG NPs group compared to the control group, while the significant increase in the hCG group compared to the control group was at 168 h. There were no significant differences between the hCG NPs and hCG groups (P ˃ 0.05).

### Hormonal and biochemical analysis

**Fig. 5:**
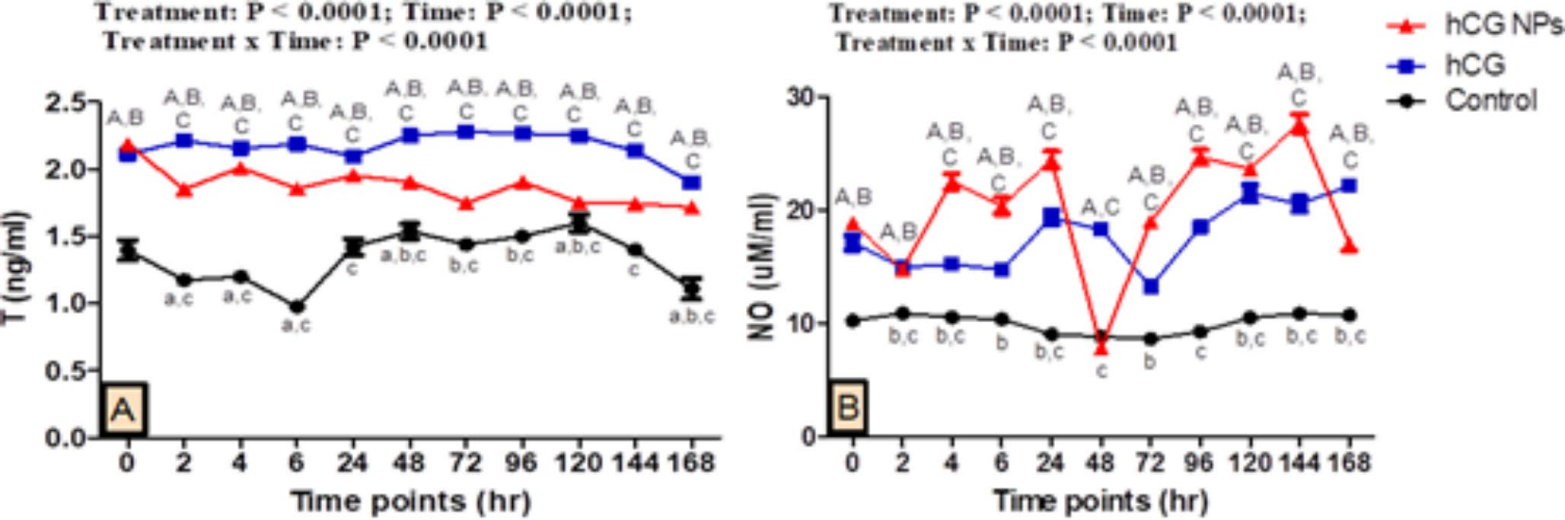
Changes in testosterone [T (**A**)] and nitric oxide concentrations [NO (**B**)] in pubescent goat bucks in the hCG NPs group (red line), hCG group (blue line), and the control group (black line) (*n* = 5, each) during different time points (hours). Values are means ± SEM. Values with superscripts A, B, and C represent significant differences between groups of hCG and control, hCG NPs and control, and hCG NPs and hCG, respectively, during the time point of the study (*P* < 0.05 for all). ^a, b, c^ Values represent the significant difference between 0 h and the indicated point within control, hCG, and hCG NPs groups, respectively

The effect of hCG NPs and hCG administration in pubescent goat bucks on testosterone and NO metabolites is presented in Fig. 5. There were treatment, time, and interaction effects (*P* < 0.0001) on the testosterone and NO concentration. Testosterone concentrations were significantly increased in the hCG group followed by the hCG NPs group in comparison with the control group. Regarding the hCG group, there was a significant increase in testosterone concentrations from 48 h to the end of the study after hCG administration compared to 0 h, and the greatest concentration was at 72 h (2.277 ± 0.006 ng/ml) compared to that in hCG NPs group (1.747 ± 0.020 ng/ml) and control group (1.44 ± 0.029 ng/ml) at this time point. Regarding the hCG NPs group, there was a significant decrease (*P* < 0.001) in testosterone concentrations at all studied time points after hCG NPs administration compared to 0 h. There were significant increases (*P* < 0.001) in the concentrations of NO in the hCG NPs and hCG groups compared to the control group. The increase in the concentrations of NO was observed in the hCG NPs group nearly at all studied time points compared to hCG and control groups except at 48 h (*P* < 0.001), there was a significant decrease (*p* < 0.001) compared to those found in the hCG group.

## Discussion

hCG is a useful diagnostic test for assessing the functional capability of the male reproductive endocrine system (Altoé et al. 2014; Samir et al. 2015) because it stimulates the Leydig cells to secrete testosterone when administered. Due to its high molecular weight and possible adverse side effects upon administration, we hypothesized that hCG injection in nanoparticle form might improve testicular function in male goats by raising TBF, testosterone levels, and testicular volume. According to our findings, the Doppler indices (RI and PI) within the STA in the hCG group and the hCG NPs group were significantly lower than in the control group. Blood flow and Doppler indices had an inverse relationship. Reduced RI and PI values are indicative of increased testicular vascular perfusion and decreased blood flow resistance, which helps to maintain the constant supply of the testis with nutrients and oxygen (Dickey 1997; Ginther 2007; Samir et al. 2024). Our findings were consistent with those reported in humans (Middleton et al. 1989), dogs (Gumbsch et al. 2002), stallions (Bollwein et al. 2008), buffalo (Abdelnaby 2022), goat bucks (Samir et al. 2015), and rats (Damber et al. 1985). Blood flow of the testis increased in the hCG and hCG NPs groups in comparison to the control one. Increased TBF following the administration of hCG or its NPs could be an attempt to counteract the detrimental effects of heat stress on blood perfusion to the testis (Samir et al. 2018; Hedia et al. 2020). To the best of our knowledge, this was the first study to use hCG in the form of NPs in goat bucks, intending to reduce the hCG dose while maintaining positive impacts on TBF and allowing treatment of more animal groups with the same conventional dose. Our findings demonstrated substantial decreases in Doppler indices (RI and PI) in the hCG NPs group when compared to the control group, indicating an increase in TBF even after administering 25% of the dose compared to the hCG group. One possible explanation for this is that nano-delivered drugs have the ability to extend the half-life of the hormone, improving its passage across blood tissue barriers such as the testicular blood barrier and cellular uptake, as well as sustaining its delivery to the target sites (Esquivel et al. 2015; Hashem and Gonzalez-Bulnes 2020).

B-mode ultrasonography has been recognized as an accurate and reproducible approach for measuring testicular volume and assessing testicular function and pubertal development (Paltiel et al. 2002; Sakamoto et al. 2008; Sarlós et al. 2013). Testicular volume estimation is important to ensure reproductive capacity because it is largely a reflection of sperm cell production (Sarlós et al. 2013). After receiving hCG or NPs, there was an increase in testicular volume in our study, which was also observed in goat bucks (Samir et al. 2015), rats (Papparella et al. 2013), and men (Kim et al. 2011). Increasing testicular volume could be attributable to the effect of hCG on the increased vascular permeability and lymph flow in the testis (Sharpe 1977; Setchell and Sharpe 1981; Damber et al. 1985), which might be a cause for increasing testicular volume. A negative correlation has been found between testicular volume and the RI and PI of the STA (Samir et al. 2015; Ribeiro et al. 2020). Decreased Doppler indices after administration of the hCG or its NPs could indicate an increase in testicular vascularity, leading to an increase in testicular perfusion, testicular vascular permeability, and interstitial fluid (IF).

Testicular echogenicity is considered a complementary noninvasive approach to assess the testicular function through the assessment of some parameters such as the PIX (Brito et al. 2012). In our study, there were significant increases in the PIX after administration of either hCG or hCG NPs. These findings might be attributed to an alteration in the TBF at this time point as previously reported (El-Shalofy et al. 2021). Furthermore, increases in the PIX could be accredited to increases in the concentration of testosterone. hCG indirectly affects testicular echogenicity by stimulating Leydig cells to produce testosterone which in turn causes changes in seminiferous tubules and interstitial cells that cause changes in echogenicity. Testosterone plays a crucial role in the testicular cell development and function that could reflected in the changes in the echogenicity of the testicular parenchyma as previously reported (Brito et al. 2012; Samir et al. 2018; Hedia et al. 2019).

Testosterone hormone is the primary androgen responsible for fetal male sexual differentiation, pubertal development, spermatogenesis maintenance, and germ cell apoptosis inhibition (Chandra et al. 2007, 2010). Our findings revealed an increase in testosterone concentration in both the hCG and hCG NPs groups compared to the control one. hCG injection caused substantial rises in testosterone concentrations at all time points of the study. This could be attributed to the direct effect of hCG on testosterone secretion through binding to the luteinizing hormone (LH) receptor, whereas in the control group, the testosterone response is secondary to LH release under the influence of gonadotropin-releasing hormone ( Ascoli et al. 2002). Biphasic testosterone response was documented in goat bucks at 2 and 96 h (Samir et al. 2015), in stallions at 1–3 and 24–72 h (Bollwein et al. 2008), and in buffalo bulls at 4–6 h (Abdelnaby 2022). The testosterone response in our study is somewhat comparable to those previously documented in goat bucks and stallions because it increased from 48 h to 96 h with the highest concentration at 72 h following hCG administration. This variation in response could be attributed to differences in animal species, animal age, hormone source, dose, and route of administration as reported in stallions (Bollwein et al. 2008), goat bucks (Samir et al. 2015), and buffalo bulls (Abdelnaby 2022). In the present study, testosterone concentration was significantly lower in the hCG NPs compared to that in the hCG-administered goat bucks. The reason behind this decline is out of the scope of this study, however, it could be related to the molecular weight of the NPs, their binding to receptors, or the reduction of the hCG dose (Roth et al. 2010).

Several investigations indicated critical functions that NO could play in the control of testicular hemodynamics and vascular tone because of its potent vasodilatory impact (Lissbrant et al. 1997; Paulis and Šimko 2007; Abdelkhalek et al. 2022). According to Lissbrant et al. (1997), the nitric oxide syntheses (NOS) system activity was reduced in basal conditions but increased in response to hCG administration. This suggests that the rise in TBF seen in hormonally stimulated testis may be linked to an increase in testicular NO synthesis. In the present research, there was an increase in NO concentrations at almost all time points after hCG administration, as previously reported (Lissbrant et al. 1997). In the hCG NPs group, there was an increase in NO concentrations at almost all time points, with a prominent decline at 48 h. The decrease in NO concentration at this time point could be attributed to an increase in free radicals, which stimulate the conversion of NO to peroxynitrite and reduce NO availability in the blood (Tsai-Turton and Luderer 2006; Hedia et al. 2020). The current study investigated the comparison of hCG and its nanoparticles on buck reproductive function as an important feature having new scientific value, which can be directly transferred as having a large practical relevance under heat stress conditions. However, the rather small group size (animals in each group, is a limitation of the study. Further research could be valuable if the experimental procedures are performed on a large scale and assess fertility-related parameters (semen quality, pregnancy diagnosis,…etc).

## Conclusion

Collectively, a single intramuscular injection of hCG or its NPs improves testicular vascularization and increases testosterone and NO concentrations and testicular volume. Nanotechnology can effectively lower the dose of hCG to its quarter with the same positive effects on testicular hemodynamics and testosterone levels. So, using NPs of hCG could be recommended for improving the reproductive performance of goat bucks under heat stress conditions and treating more animals with the same conventional dose of the hormone.

## Electronic supplementary material

Below is the link to the electronic supplementary material.

Supplementary Material 1

## Data Availability

The data that support this study are available in the article.

## References

[CR1] Abbas M, Khan MIUR, Hameed N, Rehman A, Mohsin I, Bilal M, Shahzad M (2021) Melatonin along with eCG improves fresh semen quality and plasma concentrations of melatonin and testosterone during non-breeding season in Beetal bucks. Small Rumin Res 205:106569. 10.1016/j.smallrumres.2021.106569

[CR2] Abdelkhalek KG, Badawy ABA, Fathi M, Abdelnaby EA (2022) Reproductive hormonal levels and nitric oxide levels as guides of pubertal reproductive development in relation to testicular width and hemodynamics in baladi bucks. Adv Anim Vet Sci 10(2):236–243. 10.17582/journal.aavs/2022/10.2.236.243

[CR3] Abdelnaby EA (2022) Testicular haemodynamics, plasma testosterone and oestradiol concentrations, and serum nitric oxide levels in the Egyptian buffalo bull after a single administration of human chorionic gonadotropin. Reprod Domest Anim 57(7):754–760. 10.1111/rda.14117 35352415 10.1111/rda.14117

[CR4] Ahmadi B, Lau CP, Giffin J, Santos N, Hahnel A, Raeside J, Christie H, Bartlewski P (2012) Suitability of epididymal and testicular ultrasonography and computerized image analysis for assessment of current and future semen quality in the ram. Exp Biol Med (Maywood) 237(2):186–193. 10.1258/ebm.2011.011050 22302707 10.1258/ebm.2011.011050

[CR5] Altoé PM, Tatsuo ES, Paulo DNS, Jarske R, Milagres M, Loureiro ID (2014) Effects of human chorionic gonadotropin on the normal testicular tissue of rats. Acta Cir Bras 29(05):292–298. 10.1590/S0102-86502014000500002 24863316 10.1590/s0102-86502014000500002

[CR6] Ascoli M, Fanelli F, Segaloff DL (2002) The lutropin/choriogonadotropin receptor, a 2002 perspective. Endocr Rev 23(2):141–174. 10.1210/edrv.23.2.0462 11943741 10.1210/edrv.23.2.0462

[CR7] Bansal AK, Bilaspuri GS (2011) Impacts of oxidative stress and antioxidants on semen functions. Vet Med Int 2011(1):686137. 10.4061/2011/686137 10.4061/2011/686137 PMC2943128 20871827

[CR8] Bergh A, Rooth P, Widmark A, Damber JE (1987) Treatment of rats with hCG induces inflammation-like changes in the testicular microcirculation. Reproduction 79(1):135–143. 10.1530/jrf.0.0790135 10.1530/jrf.0.0790135 3820165

[CR9] Bernabucci U, Ronchi B, Lacetera N, Nardone A (2002) Markers of oxidative status in plasma and erythrocytes of transition dairy cows during hot season. J Dairy Sci 85(9):2173–2179. 10.3168/jds.S0022-0302(02)74296-3 12362449 10.3168/jds.S0022-0302(02)74296-3

[CR11] Bollwein H, Schulze JJ, Miyamoto A, Sieme H (2008) Testicular blood flow and plasma concentrations of testosterone and total estrogen in the stallion after the administration of human chorionic gonadotropin. J Reprod Dev 54(5):335–339. 10.1262/jrd.20014 18667792 10.1262/jrd.20014

[CR10] Bollwein H, Heppelmann M, Lüttgenau J (2016) Ultrasonographic doppler use for female reproduction management. Vet Clin North Am Food Anim Pract 32(1):149–164. 10.1016/j.cvfa.2015.09.005 26922117 10.1016/j.cvfa.2015.09.005

[CR12] Brito LFC, Barth AD, Wilde RE, Kastelic JP (2012) Testicular ultrasonogram pixel intensity during sexual development and its relationship with semen quality, sperm production, and quantitative testicular histology in beef bulls. Theriogenology 78(1):69–76. 10.1016/j.theriogenology.2012.01.022 22401830 10.1016/j.theriogenology.2012.01.022

[CR13] Camela ESC, Nociti RP, Santos VJC, Macente BI, Murawski M, Vicente WRR, Bartlewski PM, Oliveira MEF (2019) Changes in testicular size, echotexture, and arterial blood flow associated with the attainment of puberty in Dorper rams raised in a subtropical climate. Reprod Domest Anim 54(2):131–137. 10.1111/rda.13213 29989218 10.1111/rda.13213

[CR15] Chandra AK, Chatterjee A, Ghosh R, Sarkar M, Chaube SK (2007) Chromium induced testicular impairment in relation to adrenocortical activities in adult albino rats. Reprod Toxicol 24(3–4):388–396. 10.1016/j.reprotox.2007.07.009 17822870 10.1016/j.reprotox.2007.07.009

[CR14] Chandra AK, Chatterjee A, Ghosh R, Sarkar M (2010) Vitamin E-supplementation protect chromium (VI)-induced spermatogenic and steroidogenic disorders in testicular tissues of rats. Food Chem Toxicol 48(3):972–979. 10.1016/j.fct.2010.01.008 20079796 10.1016/j.fct.2010.01.008

[CR16] Damber JE, Bergh A, Daehlin L (1985) Testicular blood flow, vascular permeability, and testosterone production after stimulation of unilaterally cryptorchid adult rats with human chorionic gonadotropin. Endocrinol 117(5):1906–1913. 10.1210/endo-117-5-1906 10.1210/endo-117-5-1906 2864238

[CR17] Dangi SS, Dangi SK, Chouhan VS, Verma MR, Kumar P, Singh G, Sarkar M (2016) Modulatory effect of betaine on expression dynamics of HSPs during heat stress acclimation in goat (Capra hircus). Gene 575(2):543–550. 10.1016/j.gene.2015.09.031 26390816 10.1016/j.gene.2015.09.031

[CR18] Dickey RP (1997) Doppler ultrasound investigation of uterine and ovarian blood flow in infertility and early pregnancy. Hum Reprod Update 3(5):467–503. 10.1093/humupd/3.5.467 9528912 10.1093/humupd/3.5.467

[CR19] El-Shalofy A, Hedia M, Kastelic J (2021) Melatonin improves testicular haemodynamics, echotexture and testosterone production in Ossimi rams during the breeding season. Reprod Domest Anim 56(11):1456–1463. 10.1111/rda.14010 34459033 10.1111/rda.14010

[CR22] El-Sherbiny HR, Fathi M, Samir H, Abdelnaby EA (2022a) Supplemental dietary curcumin improves testicular hemodynamics, testosterone levels, and semen quality in Baladi bucks in the nonbreeding season. Theriogenology 188:100–107. 10.1016/j.theriogenology.2022.05.020 35688039 10.1016/j.theriogenology.2022.05.020

[CR20] El-Sherbiny HR, Abdelnaby EA, El-Shahat KH, Salem NY, Ramadan ES, Yehia SG, Fathi M (2022b) Coenzyme Q10 Supplementation enhances testicular volume and hemodynamics, reproductive hormones, sperm quality, and seminal antioxidant capacity in goat bucks under summer hot humid conditions. Vet Res Commun 46(4):1245–1257. 10.1007/s11259-022-09991-8 36048337 10.1007/s11259-022-09991-8 PMC9684261

[CR21] El-Sherbiny HR, El-Shalofy AS, Samir H (2022c) Exogenous L-carnitine administration ameliorates the adverse effects of Heat stress on testicular hemodynamics, echotexture, and total antioxidant capacity in rams. Front Vet Sci 9:860771. 10.3389/fvets.2022.860771 35464382 10.3389/fvets.2022.860771 PMC9019560

[CR23] El-Tarabany MS, El-Tarabany AA, Atta MA (2017) Physiological and lactation responses of Egyptian dairy Baladi goats to natural thermal stress under subtropical environmental conditions. Int J Biometeorol 61:61–68. 10.1007/s00484-016-1191-2 27225437 10.1007/s00484-016-1191-2

[CR24] Esquivel R, Juárez J, Almada M, Ibarra J, Valdez MA (2015) Synthesis and characterization of new thiolated chitosan nanoparticles obtained by ionic gelation method. Int J Polym Sci 2015(1):502058. 10.1155/2015/502058

[CR25] Fadl AM, Abdelnaby EA, El-Sherbiny HR (2022) Supplemental dietary zinc sulfate and folic acid combination improves testicular volume and hemodynamics, testosterone levels and semen quality in rams under heat stress conditions. Reprod Domest Anim 57(6):567–576. 10.1111/rda.14096 35147249 10.1111/rda.14096

[CR26] Ginther OJ (2007) Ultrasonic imaging and animal reproduction: color-Doppler ultrasonography—Book 4. Equiservices Publishing, Cross plains, pp 7–81

[CR27] Gloria A, Di Francesco L, Marruchella G, Robbe D, Contri A (2020) Pulse-wave Doppler pulsatility and resistive indexes of the testicular artery increase in canine testis with abnormal spermatogenesis. Theriogenology 158:454–460. 10.1016/j.theriogenology.2020 33049570 10.1016/j.theriogenology.2020.10.015

[CR28] Gumbsch P, Holzmann A, Gabler C (2002) Colour-coded duplex sonography of the testes of dogs. Vet Rec 151(5):140–144. 10.1136/vr.151.5.140 12199432 10.1136/vr.151.5.140

[CR29] Günzel-Apel AR, Möhrke C, Nautrup C (2001) Colour‐coded and pulsed Doppler sonography of the canine testis, epididymis and prostate gland: physiological and pathological findings. Reprod Domest Anim 36(5):236–240 11885739 10.1046/j.1439-0531.2001.00288.x

[CR30] Hansen PJ (2009) Effects of heat stress on mammalian reproduction. Philos Trans R Soc Lond B Biol Sci 364(1534):3341–33450. 10.1098/rstb.2009.0131 19833646 10.1098/rstb.2009.0131 PMC2781849

[CR32] Hashem NM, Gonzalez-Bulnes A (2020) State-of-the-art and prospective of nanotechnologies for smart reproductive management of farm animals. Animals 10(5):840. 10.3390/ani10050840 32414174 10.3390/ani10050840 PMC7278443

[CR31] Hashem A, Hammam A (2009) Impact of birth season on puberty in female and male kids of Shami goat in north Sinai, Egypt. J Anim Poult Prod 34(1):139–150. 10.21608/JAPPMU.2009.112336

[CR33] Hashem NM, Sallam SM (2020) Reproductive performance of goats treated with free gonadorelin or nanoconjugated gonadorelin at Estrus. Domest Anim Endocrinol 71:106390. 10.1016/j.domaniend.2019.106390 31731249 10.1016/j.domaniend.2019.106390

[CR34] Hassanein EM, Hashem NM, El-Azrak KEDM, Gonzalez-Bulnes A, Hassan GA, Salem MH (2021) Efficiency of GnRH–loaded chitosan nanoparticles for inducing LH secretion and fertile ovulations in protocols for artificial insemination in rabbit does. Animals 11(2):440. 10.3390/ani11020440 33567711 10.3390/ani11020440 PMC7914616

[CR36] Hedia MG, El-Belely MS, Ismail ST, El-Maaty AMA (2019) Monthly changes in testicular blood flow dynamics and their association with testicular volume, plasma steroid hormones profile and semen characteristics in rams. Theriogenology 123:68–73. 10.1016/j.theriogenology.2018.09.032 30292858 10.1016/j.theriogenology.2018.09.032

[CR35] Hedia MG, El-Belely MS, Ismail ST, Abo El‐Maaty AM (2020) Seasonal variation in testicular blood flow dynamics and their relation to systemic and testicular oxidant/antioxidant biomarkers and androgens in rams. Reprod Domest Anim 55(7):861–869. 10.1111/rda.13696 32374490 10.1111/rda.13696

[CR37] Kay GW, Grobbelaar JA, Hattingh J (1992) Effect of surgical restriction of growth of the testicular artery on testis size and histology in bulls. Reproduction 96(2):549–553 10.1530/jrf.0.0960549 1339835

[CR38] Kendall P, Webster J (2009) Season and physiological status affects the circadian body temperature rhythm of dairy cows. Livest Sci 125:155–160. 10.1016/j.livsci.2009.04.004

[CR39] Kim SO, Ryu KH, Hwang IS, Jung SI, Oh KJ, Park K (2011) Penile growth in response to human chorionic gonadotropin (HCG) treatment in patients with idiopathic hypogonadotrophic hypogonadism. Chonnam Med J 47(1):39–42. 10.4068/cmj.2011.47.1.39 22111055 10.4068/cmj.2011.47.1.39 PMC3214853

[CR40] Lissbrant E, Löfmark U, Collin O, Bergh A (1997) Is nitric oxide involved in the regulation of the rat testicular vasculature? Biol Reprod 56(5):1221–1227. 10.1095/biolreprod56.5.1221 9160722 10.1095/biolreprod56.5.1221

[CR41] Lu CD (1989) Effects of heat stress on goat production. Small Rumin Res 2(2):151–162. 10.1016/0921-4488(89)90040-0

[CR42] Maquivar MG, Smith SM, Busboom JR (2021) Reproductive management of rams and ram lambs during the pre-breeding season in US sheep farms. Animals 11(9):2503 34573469 10.3390/ani11092503 PMC8471565

[CR43] Marai IFM, Abou-Fandoud EI, Daader AH, Abu-Ella AA (2002) Reproductive doe traits of the nubian (Zaraibi) goats in Egypt. Small Rumin Res 46(2–3):201–205. 10.1016/S0921-4488(02)00195-5

[CR44] Maroto-Morales A, García-Álvarez O, Ramón M, Martínez-Pastor F, Fernández-Santos MR, Soler AJ, Garde JJ (2016) Current status and potential of morphometric sperm analysis. Asian J Androl 18(6):863–870. 10.4103/1008-682X.187581 27678465 10.4103/1008-682X.187581 PMC5109877

[CR45] Middleton WD, Thorne DA, Melson GL (1989) Color Doppler ultrasound of the normal testis. Am J Roentgenol 152(2):293–297. 10.2214/ajr.152.2.293 2643264 10.2214/ajr.152.2.293

[CR46] Paltiel HJ, Diamond DA, Di Canzio J, Zurakowski D, Borer JG, Atala A (2002) Testicular volume: comparison of orchidometer and US measurements in dogs. Radiology 222:114–119. 10.1148/radiol.2221001385 11756714 10.1148/radiol.2221001385

[CR47] Pamungkas FA, Sianturi RSG, Wina E, Kusumaningrum DA (2016) Chitosan nanoparticle of hCG (Human Chorionic Gonadotrophin) hormone in increasing induction of dairy cattle ovulation. JITV 21(1):34–40. 10.14334/jitv.v21i1.1343

[CR48] Papparella A, Nino F, Noviello C, Romano M, Papparella S, Pa-ciello O, Sinisi AA (2013) Morphologic changes due to human chorionic gonadotropin in the rat testis: role of vascular endothelial growth factor. Open J Pediatr 3:85–91. 10.4236/ojped.2013.32016

[CR49] Paul C, Murray AA, Spears N, Saunders PT (2008) A single, mild, transient scrotal heat stress causes DNA damage, subfertility and impairs formation of blastocysts in mice. Reproduction 136(1):73–84. 10.1530/REP-08-0036 18390691 10.1530/REP-08-0036

[CR50] Paulis L, Šimko F (2007) Blood pressure modulation and cardiovascular protection by melatonin: potential mechanisms behind. Physiol Res 56(6). 10.33549/physiolres.931236 10.33549/physiolres.931236 18197748

[CR51] Pozor A, McDonnell M (2004) Color Doppler ultrasound evaluation of testicular blood flow in stallions. Theriogenology 61:799–810. 10.1016/S0093-691X(03)00227-9 14757466 10.1016/s0093-691x(03)00227-9

[CR53] Pozor MA, Macpherson ML, Troedsson MHT, Verstegen J (2006) Effect of a single administration of human chorionic gonadotropin (hCG) on testicular blood flow in stallions. Anim Reprod Sci 94(1–4):146–147

[CR52] Pozor M, Morrissey H, Albanese V, Khouzam N, Deriberprey A, Macpherson ML, Kelleman AA (2017) Relationship between echotextural and histomorphometric characteristics of stallion testes. Theriogenology 99:134–145. 10.1016/j.theriogenology.2017.05.031 28708494 10.1016/j.theriogenology.2017.05.031

[CR54] Rao CV, Alsip NL (2001) Use of the rat model to study hCG/LH effects on uterine blood flow. Semin Reprod Med 19(1):75–86. 10.1055/s-2001-13914 11394208 10.1055/s-2001-13914

[CR55] Ribeiro DLDS, Santos LS, França IG, Pereira HG, Lima JDS, Miranda BDB, Junior JRST (2020) The use of Doppler ultrasound as a potential fertility predictor in male goats. Turk J Vet Anim Sci 44(5):1115–1124. 10.3906/vet-2003-135

[CR56] Roth MY, Page ST, Lin K, Anawalt BD, Matsumoto AM, Snyder CN, Marck BT, Bremner WJ, Amory JK (2010) Dose-dependent increase in intratesticular testosterone by very low-dose human chorionic gonadotropin in normal men with experimental gonadotropin deficiency. J Clin Endocrinol Metab 95(8):3806–3813. 10.1210/jc.2010-0360 20484472 10.1210/jc.2010-0360 PMC2913032

[CR57] Sakamoto H, Ogawa Y, Yoshida H (2008) Relationship between testicular volume and testicular function: comparison of the Prader orchidometric and ultrasonographic measurements in patients with infertility. Asian J Androl 10(2):319–324. 10.1111/j.1745-7262.2008.00340 18097521 10.1111/j.1745-7262.2008.00340.x

[CR58] Saleh M, Shahin M, Wuttke W, Gauly M, Holtz W (2012) Pharmacokinetics of human chorionic gonadotropin after im administration in goats (Capra hircus). Reproduction 144(1):77. 10.1530/REP-12-0093 22573828 10.1530/REP-12-0093

[CR61] Samir H, Sasaki K, Ahmed E, Karen A, Nagaoka K, El Sayed M, Taya K, Watanabe G (2015) Effect of a single injection of gonadotropin-releasing hormone (GnRH) and human chorionic gonadotropin (hCG) on testicular blood flow measured by color doppler ultrasonography in male Shiba goats. J Vet Med Sci 77(5):549–556. 10.1292/jvms.14-0633 25715956 10.1292/jvms.14-0633 PMC4478734

[CR60] Samir H, Nyametease P, Nagaoka K, Watanabe G (2018) Effect of seasonality on testicular blood flow as determined by color Doppler ultrasonography and hormonal profiles in Shiba goats. Anim Reprod Sci 197:185–192. 10.1016/j.anireprosci.2018.08.027 30166078 10.1016/j.anireprosci.2018.08.027

[CR59] Samir H, Nyametease P, Elbadawy M, Nagaoka K, Sasaki K, Watanabe G (2020) Administration of melatonin improves testicular blood flow, circulating hormones, and semen quality in Shiba goats. Theriogenology 146:111–119. 10.1016/j.theriogenology.2020.01.053 32078960 10.1016/j.theriogenology.2020.01.053

[CR62] Samir H, Swelum AA, Farag A, El-Sherbiny HR (2024) Emotional temperaments in advanced pregnant goats and its relationship with the feto-maternal blood flow and placentome echotexture. Vet Res Commun 48(3):1545–1561. 10.1007/s11259-024-10330-2 38379058 10.1007/s11259-024-10330-2 PMC11147941

[CR63] Sarlós P, Egerszegi I, Balogh O, Molnár A, Cseh S, Rátky J (2013) Seasonal changes of scrotal circumference, blood plasma testosterone concentration and semen characteristics in Racka rams. Small Rumin Res 111(1–3):90–95. 10.1016/j.smallrumres.2012.11.036

[CR64] Setchell BP (1990) Local control of testicular fluids. Reprod Fertil Dev 2(3):291–309. 10.1071/RD9900291 2201064 10.1071/rd9900291

[CR65] Setchell BP (2006) The effects of heat on the testes of mammals. Anim Reprod 3(2):81–91

[CR66] Setchell BP, Sharpe RM (1981) Effect of injected human chorionic gonadotrophin on capillary permeability, extracellular fluid volume and the flow of lymph and blood in the testes of rats. J Endocrinol 91(2):245–254. 10.1677/joe.0.0910245 7299325 10.1677/joe.0.0910245

[CR67] Shahat AM, Rizzoto G, Kastelic JP (2020) Amelioration of heat stress-induced damage to testes and sperm quality. Theriogenology 158:84–96. 10.1016/j.theriogenology.2020.08.034 32947064 10.1016/j.theriogenology.2020.08.034

[CR68] Sharpe RM (1977) Relationship between testosterone, fluid content and luteinizing hormone receptors in the rat testis. Biochem Biophys Res Commun 75(3):711–717. 10.1016/0006-291X(77)91530-3 193500 10.1016/0006-291x(77)91530-3

[CR69] Shi QJ, Lei ZM, Rao CV, Lin J (1993) Novel role of human chorionic gonadotropin in differentiation of human cytotrophoblasts. Endocrinol 132(3):1387–1395. 10.1210/en.132.3.1387 10.1210/endo.132.3.7679981 7679981

[CR70] Strina A, Corda A, Nieddu S, Solinas G, Lilliu M, Zedda MT, Ledda S (2016) Annual variations in resistive index (RI) of testicular artery, volume measurements and testosterone levels in bucks. Comp Clin Path 25:409–413. 10.1007/s00580-015-2199-4

[CR71] Tibary A, Boukhliq R, El Allali K (2018) Ram and Buck breeding soundness examination. Rev Maroc Des Sci Agron et Vétérinaires 6(2):241–255

[CR72] Tsai-Turton M, Luderer U (2006) Opposing effects of glutathione depletion and follicle-stimulating hormone on reactive oxygen species and apoptosis in cultured preovulatory rat follicles. Endocrinol 147(3):1224–1236. 10.1210/en.2005-1281 10.1210/en.2005-1281 16339198

[CR73] Varughese EE, Brar PS, Dhindsa SS (2013) Uterine blood flow during various stages of pregnancy in dairy buffaloes using transrectal Doppler ultrasonography. Anim Reprod Sci 140(1–2):34–39. 10.1016/j.anireprosci.2013.05.011 23773326 10.1016/j.anireprosci.2013.05.011

[CR74] Zygmunt M, Herr F, Keller-Schoenwetter S, Kunzi-Rapp K, Münstedt K, Rao CV, Preissner KT (2002) Characterization of human chorionic gonadotropin as a novel angiogenic factor. J Clin Endocrinol Metab 87(11):5290–5296. 10.1210/jc.2002-020642 12414904 10.1210/jc.2002-020642

