# Supplementary material for: Effect of administration of nanoparticles of human chorionic gonadotropin on testicular hemodynamics, testicular volume, testicular echotexture, and circulating testosterone and nitric oxide in pubescent goat bucks under heat stress conditions
Source: Vet Res Commun. 2025 Jan 15;49(2):78. doi: 10.1007/s11259-024-10634-3 (PMC11735597; doi:10.1007/s11259-024-10634-3)
Supplement: Supplementary file 1 — Supplementary Material 1 [file 11259_2024_10634_MOESM1_ESM.docx]

**hCG NANO-EMULSION**

**Material and Methods**

**Materials:**

EIPICO Company (Co.) supplied human chorionic gonadotropin (hCG), Sigma-Aldrich Co. supplied non-ionic surfactant (Tween 80 and Tween 20) and NineLife Co. supplied soybean oil.

**Preparation of hCG Nano-emulsion:**

**Method of preparation:**

The oil phase (Soybean) was slowly added to 50 ml of deionized water after 8 mg of hCG was mixed with 25 ml of tween 20 and 25 ml of tween 80 with a Probe homogenizer (LK Lab, Korea). The method mentioned by **Silva et al. (2011)** was followed to prepare a nano-emulsion.

**Method of characterization:**

A thorough evaluation of the physical and chemical properties of hCG nano-emulsion was performed to assess its biological capabilities. The specimen has been indexed, identified, and characterized microscopically. Using transmission electron microscopy (TEM) ( Jeol, JEM-2100 high-resolution, Japan), hCG nano-emulsion shape and surface topography were determined using AFM (atomic force microscopy) (Agilent, USA). Using DLS (dynamic light scattering) (Malvern, UK), we were able to ascertain the size and zeta potential of the particles to estimate their ability to disperse in fluids.

**Results**

**Characterization**

The results of characterization techniques are shown in Figure (1).

A TEM image of the hCG nano-emulsion as shown in Fig (1. A) revealed that the particles were spherical to subspherical with a maximum thickness of 10-30 nm. Furthermore, it was found that hCG nano-emulsion does not tend to agglomerate in certain regions with homogeneous dispersed matrix as evidenced by AFM images as shown in in fig (1. B). The results of zeta size showed that the average particle size of synthesized hCG nano-emulsion is 27.8 ± 0.2 nm as shown in in fig (1. C). Results of zeta potential showed -32.5 ± 0.01 mV as shown in Fig (1. D). Therefore, based on the results, it can be concluded that the high zeta potential of the synthesized nano-emulsion has a direct effect on the colloidal stability in water, as it is derived from the high bioactivity of the hCG nano-emulsion.

| 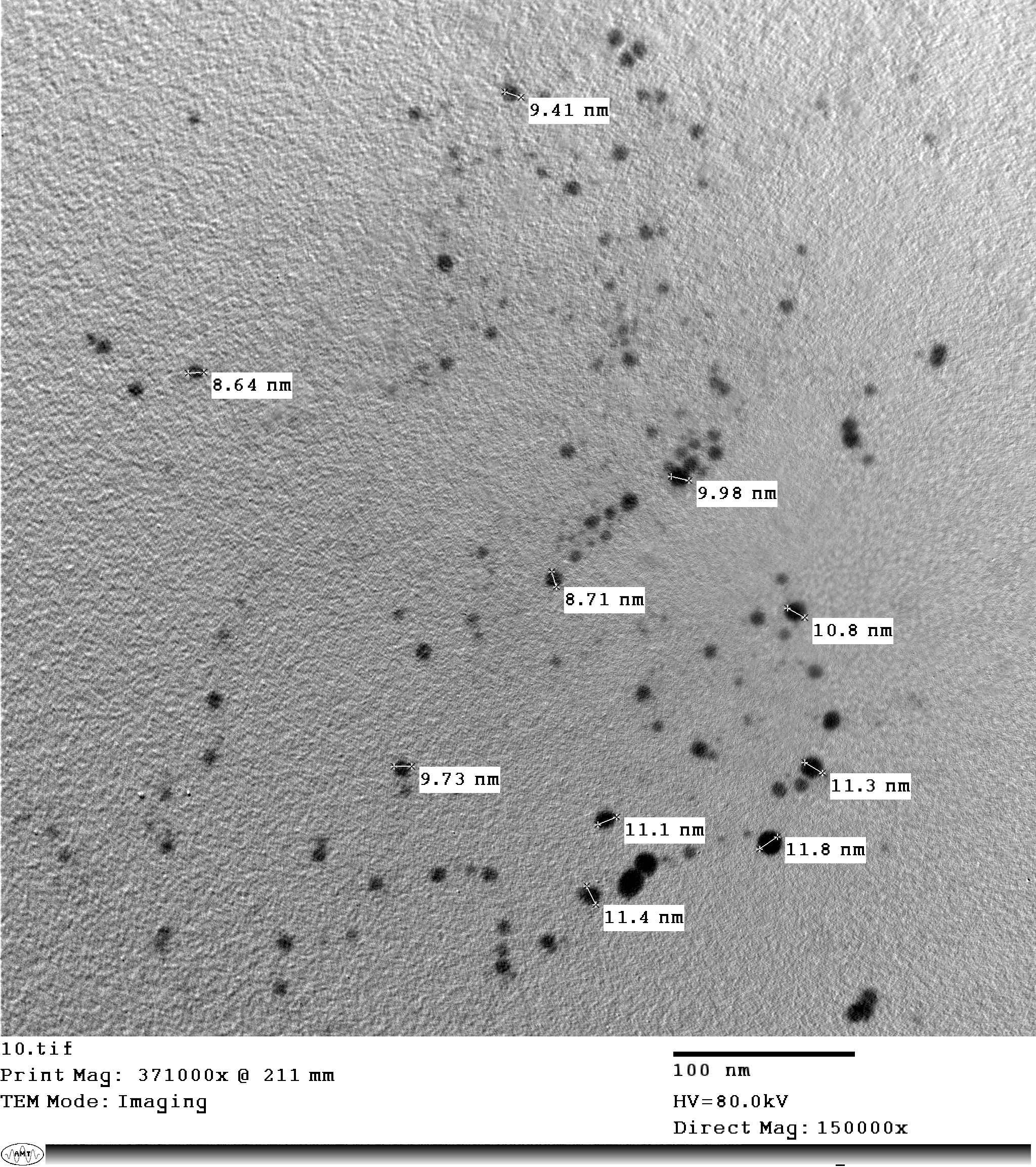  **(a)** | 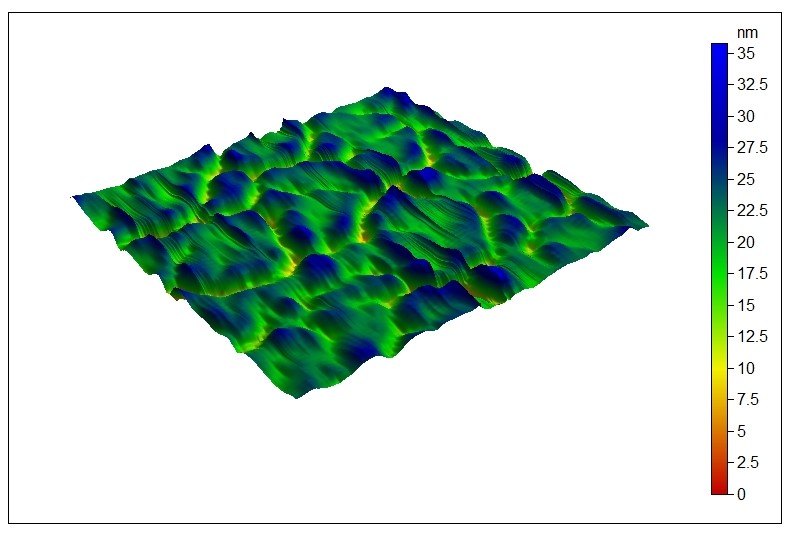  **(b)** |
| --- | --- |
| 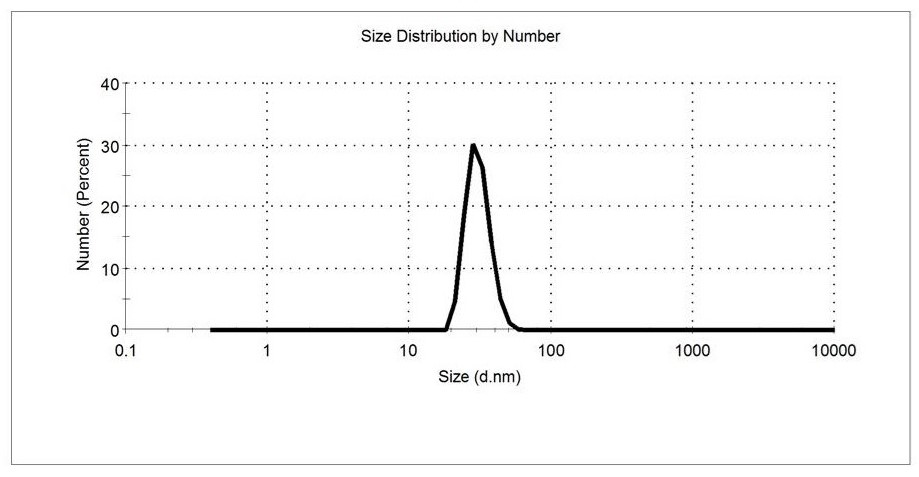  **(c)** | 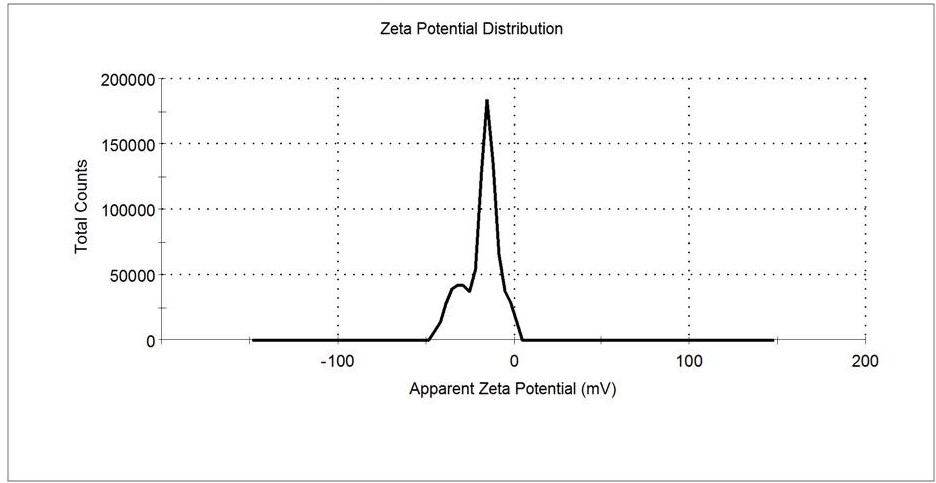  **(d)** |

**Figure (1)**: illustrated A) TEM B) AFM C) Zeta size and D) Zeta potential of **hCG nano-emulsion**.

**References**

Silva HD, Cerqueira MA, Souza BW, Ribeiro C, Avides MC, Quintas MA, Vicente AA (2011) Nanoemulsions of β-carotene using a high-energy emulsification–evaporation technique. Journal of Food Engineering 102(2), 130-135.‏ https://doi.org/10.1016/j.jfoodeng.2010.08.005
